# Sinus Tachycardia Following COVID-19 and Its Implications

**DOI:** 10.7759/cureus.57320

**Published:** 2024-03-31

**Authors:** Borna Amir-Kabirian, Frank H Annie, Morgan Koontz, Rayan Ihle

**Affiliations:** 1 Internal Medicine, CAMC Institute for Academic Medicine, Charleston, USA; 2 Cardiology, CAMC Institute for Academic Medicine, Charleston, USA; 3 Critical Care Medicine, CAMC Institute for Academic Medicine, Charleston, USA

**Keywords:** sars-cov-2, vaccination, sinus tachycardia, long covid, covid-19

## Abstract

Background

Within the cardiovascular system, sinus tachycardia has been a noted finding in patients with post-COVID-19 syndrome (symptoms persisting beyond 12 weeks post-infection). To better understand post-COVID-19 tachycardia, we examined the prevalence of sinus tachycardia 12-16 weeks after diagnosis of SARS-COV-2 infection and its correlation with intensive care utilization, ventilator use, and mortality in vaccinated and unvaccinated patients.

Methods

We identified adult patients in the TriNetX COVID-19 Research Network with confirmed SARS-COV-2 diagnosis from January 20th, 2020, to February 14th, 2022, and sinus tachycardia 12-16 weeks after diagnosis. Two cohorts were created: patients who developed tachycardia 12 weeks after initial diagnosis and patients without tachycardia. The tachycardia cohort was divided further based on vaccination status.

Results

Of 1,363,907 patients included, 30,705 (2.2%) developed tachycardia. The patients with tachycardia had more comorbidities. Using propensity score matching (PSM), two cohorts of 30,702 were created. The SARS-COV-2 tachycardic cohort had higher mortality (5.1% vs 2.1%, p<0.001), critical care utilization (5.8% vs 2.2%, p<0.001), and ventilator use (1.8% vs 0.5%, p<0.001). Out of 22,878 patients with persistent tachycardia and recorded vaccination status, 14,840 (65%) were not vaccinated. Mortality (5.9% vs 2.3%, p<0.001), critical care utilization (8.3% vs 3.6%, p<0.001), and ventilator use (3.8% vs 0.6%, p<0.001) were higher in the non-vaccinated patients compared with the vaccinated patients after PSM.

Conclusion

The prevalence of persistent tachycardia after SARS-COV-2 infection is notable at 2.2%. Patients with persistent tachycardia have higher mortality rates and demonstrate greater healthcare utilization at one year compared to patients without persistent tachycardia, particularly if unvaccinated.

## Introduction

Coronavirus disease 2019 (COVID-19) is caused by severe acute respiratory syndrome coronavirus 2 (SARS-COV-2). This highly contagious disease has resulted in over 700 million cases and nearly seven million deaths worldwide as of February 2023 [1]. After contracting the virus, many of the symptoms manifest in the respiratory system, including cough and shortness of breath. In some patients, these symptoms have lasting effects. Post-COVID syndrome is defined by symptoms of COVID-19 that persist for longer than 12 weeks after infection [2].

Post-COVID syndrome affects 10-35% of patients with a history of COVID-19 and can affect as many as 85% of patients who are hospitalized [3]. These symptoms typically include fatigue, dyspnea, or olfactory dysfunction. Sinus tachycardia has also been noted as a cardiovascular manifestation of post-COVID syndrome, with a prevalence as high as 20% [4]. The underlying pathophysiology of post-COVID tachycardia is not fully understood currently, but it is hypothesized that inflammatory cytokines are released during the period of infection that can affect cardiac ion channels and trigger arrhythmias [5]. After recovery from the initial infection, proposed mechanisms for persistent sinus tachycardia involve autonomic dysregulation and neuronal cell invasion using cell receptors [4,6].

Vaccines were developed and administered to combat COVID-19 in early 2022. As of July 2022, there were over 13 billion doses of vaccinations administered worldwide [1]. While data has suggested a decrease in hospitalizations and mortality among vaccinated individuals, the effects of vaccination on post-COVID syndrome, including post-COVID tachycardia, have not been extensively studied [7]. In fact, currently, there is limited data supporting the notion that COVID-19 can result in prolonged sinus tachycardia continuing for more than 12 weeks post-infection.

To better understand post-COVID tachycardia, we examined the prevalence of sinus tachycardia 12-16 weeks after diagnosis of SARS-COV-2 infection and its correlation with intensive care utilization, ventilator use, and mortality in vaccinated and unvaccinated patients. This abstract was previously presented in the American Thoracic Society 2023 as an oral presentation in the COVID severity, outcomes and disparities symposium.

## Materials and methods

Data source

TriNetX is a global federated health research network providing access to deidentified data (diagnoses, procedures, medications, laboratory data, and genomic information) from the electronic medical records of patients cared for predominantly in large healthcare organizations. The TriNetX COVID-19 Research Network, which comprises 76 healthcare organizations globally, provides the largest COVID-19 dataset currently available. The Institutional Review Board at Charleston Area Medical Center, Charleston West Virginia (WV) granted approval for the study before study initiation. This research is also supported by the National Institute of General Medical Sciences (2U54GM104942-02).

Study population and endpoints

To identify patients for this retrospective cohort study, we queried the TriNetX research network to select adult patients (age ≥18 years) with a confirmed diagnosis of COVID-19 from January 20th, 2020, to February 14th, 2022. Hospitalized patients were identified as COVID-19 positive if they had a billable code for COVID-19 and had any positive laboratory evidence of the infection in the past. We then determined whether or not these patients experienced sinus tachycardia 12-16 weeks after infection. Tachycardia was defined as any incidence of an average heart rate above 100 bpm documented in the database while hospitalized. Patients were excluded from this analysis if they had a history of cardiac arrhythmias, valvular dysfunction, acute myocardial infarction, acute stroke, anemia, hyperthyroidism, adrenal insufficiency, shock, or pheochromocytoma. The patients were divided into two cohorts: patients who developed tachycardia 12-16 weeks after the initial diagnosis of SARS-COV-2 and patients without a diagnosis of tachycardia. Two matched cohorts were then created to evaluate outcomes using propensity score matching (PSM) to account for potential confounding factors of demographics and co-morbidities. Outcomes for mortality, utilization of critical care services (defined as admission to the intensive care unit), and ventilator use (defined as the use of billing codes for mechanical ventilator management), were assessed at one year.

Within the cohort of patients with persistent tachycardia after SARS-COV-2 infection, a sub-analysis was conducted to compare outcomes of those who completed the primary series of COVID-19 vaccination with patients who did not receive the vaccine or did not complete the full recommended dose. Completion of the primary series was defined as two doses of Pfizer or Moderna vaccines, and one dose for Johnson & Johnson, AstraZeneca, Janssen, or Novavax vaccines.

The primary aim of this study was to evaluate the prevalence of persistent sinus tachycardia following SARS-COV-2 infection. The secondary aims were to compare the difference in healthcare utilization among individuals with persistent tachycardia following SARS-COV-2 infection and investigate the effects of vaccination on outcomes, including mortality, ICU admission, and ventilator usage in patients with confirmed SARS-COV-2 tachycardia.

Statistical analyses

Descriptive statistics were presented as frequencies with percentages for categorical variables and as mean ± SD for continuous measures. Baseline characteristics were compared using a Pearson chi-squared test for categorical variables and an independent sample t-test for continuous variables. To account for differences in baseline characteristics between the two groups, a PSM model was developed using logistic regression to derive two well-matched groups for comparative outcome analysis. Variables included in the PSM model included age, race, and key comorbidities (hypertension, diabetes, chronic obstructive lung disease, heart failure, obesity, nicotine dependence, history of stroke, and body mass index (BMI)). The TriNetX program uses logistic regression to obtain the listed propensity scores within each covariate selected using the Python libraries NumPy and sklearn (Python Software Foundation, Wilmington, DE). The platform also runs PSM in R code to compare and verify the results of the outputs. The final step in verification uses a nearest-neighbor matching algorithm with a tolerance level of 0.01, and the difference between their propensity scores must not be greater than 0.1. All-cause mortality was displayed in the PSM cohorts using the Kaplan-Meier method, and the statistical significance of the differences between the two groups was assessed using the log-rank test.

## Results

A total of 1,363,907 patients who met the inclusion criteria were studied. Among them, 30,705 (2.2%) developed tachycardia 12-16 weeks after SARS-COV-2 diagnosis, while 1,333,202 (97.8%) did not. Before PSM, patients with persistent tachycardia had higher rates of comorbidities compared with patients who did not develop tachycardia, including higher rates of hypertension (40.1% vs 21.5%, p<0.001), diabetes (24.5% vs 10.7%, p<0.001), and nicotine dependence (20.0% vs 7.6%, p<0.001). PSM created two well-matched cohorts with 30,702 subjects in each group. The demographic characteristics, comorbidities, and BMI of the patients with persistent COVID-19 tachycardia and the control patients before and after PSM are presented in Table 1. After PSM, the mean patient age was 43.2 years, with females representing more than half at 56.9% and Caucasians representing the majority (60.9%). 

**Table 1:** Baseline characteristics of study subjects with and without persistent tachycardia BMI, body mass index; CAD, coronary artery disease; COPD, chronic obstructive pulmonary disease; TIA, transient ischemic attack

	Before matching			After matching		
	COVID-19 with tachycardia (n=30,705)	COVID-19 without tachycardia (n=1,333 202)	P-value	COVID-19 with tachycardia (n=30,702)	COVID-19 without tachycardia (n=30,702)	P-value
Age at index						
Mean ± SD	43.2 ± 16.6	45.6 ± 17.2	<0.001	43.2 ± 16.6	43.2 ± 16.6	0.815
Sex						
Female	17,481 (56.9%)	729,596 (54.7%)	<0.001	17,479 (56.9%)	17,478 (56.9%)	0.993
Male	13,217 (43.0%)	583,156 (43.7%)	0.015	13,216 (43.0%)	13,216 (43.0%)	1
Race (%)						
Caucasian	18,705 (60.9%)	766,701 (57.5%)	<0.001	18,703 (60.9%)	18,711 (60.9%)	0.947
Hispanic	4,443 (14.5%)	126,515 (9.5%)	<0.00	4,441 (14.5%)	4,454 (14.5%)	0.882
African American	6,895 (22.5%)	197,348 (14.8%)	<0.001	6,894 (22.5%)	6,901 (22.5%)	0.946
Asian	566 (1.8%)	30,616 (2.3%)	<0.001	566 (1.8%)	559 (1.8%)	0.833
Comorbidities (%)						
Hypertension	12,318 (40.1%)	287,092 (21.5%)	<0.001	12,315 (40.1%)	12,336 (40.2)	0.863
CAD without angina	2,128 (6.9%)	49,446 (3.7%)	<0.001	2,127 (6.9%)	2,151 (7.0%)	0.704
Heart failure	2,190 (7.1%)	30,300 (2.3%)	<0.001	2,187 (7.1%)	2,171 (7.1%)	0.801
COPD	2,642 (8.6%)	39,636 (3.0%)	<0.001	2,639 (8.6%)	2,602 (8.5%)	0.593
Diabetes mellitus	7,536 (24.5%)	142,111 (10.7%)	<0.001	7,533 (24.5%)	7,539 (24.6%)	0.955
Nicotine dependence	6,155 (20.0%)	101,980 (7.6%)	<0.001	6,152 (20.0%)	6,162 (20.1%)	0.920
History of TIA	358 (1.8%)	9,336 (0.7%)	<0.001	358 (1.2%)	331 (1.1%)	0.301
BMI						
Mean ± SD	30.4 ± 7.7	29.7 ± 7.0	<0.001	30.4 ± 7.7	30.4 ± 7.5	0.939

**Figure 1:**
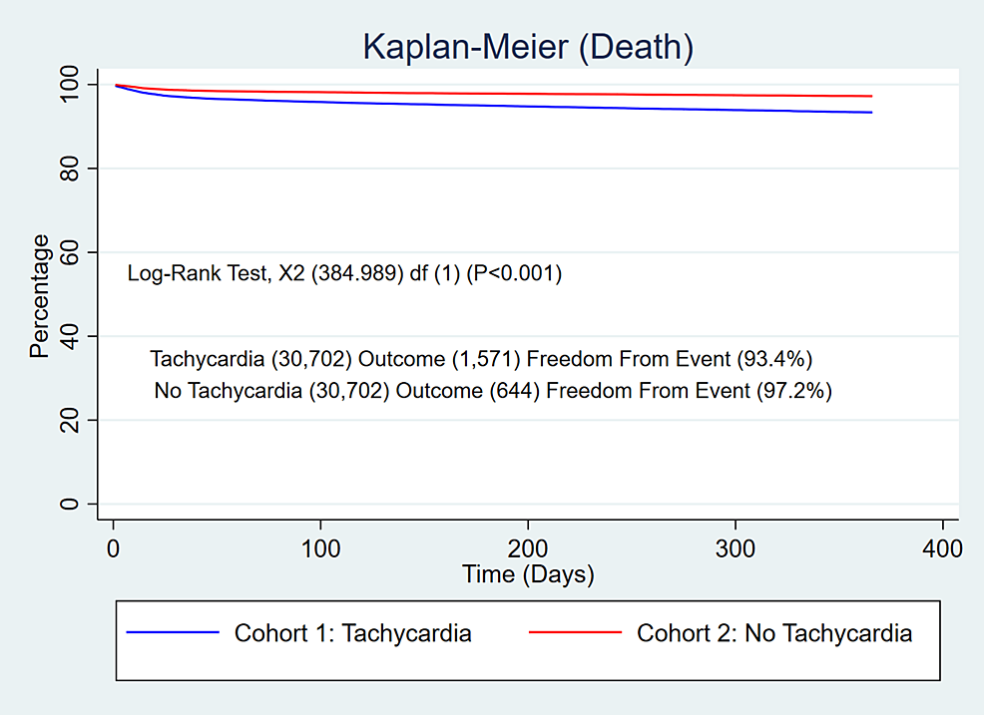
Kaplan-Meier curve of risk of mortality within one year of infection in patients with persistent tachycardia after SARS-COV-2 infection compared with the SARS-COV-2 control group without persistent tachycardia after PSM. Log-rank test: p<0.001 PSM, propensity score matching

**Figure 2:**
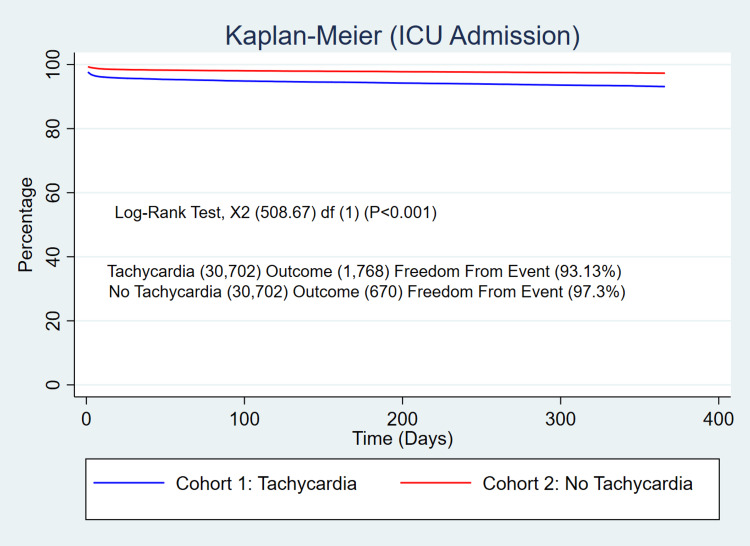
Risk of the use of critical care services/ICU admission in patients with persistent tachycardia after SARS-COV-2 infection compared with the SARS-COV-2 control group without persistent tachycardia after PSM. Log-rank test: p<0.001 PSM, propensity score matching

**Figure 3:**
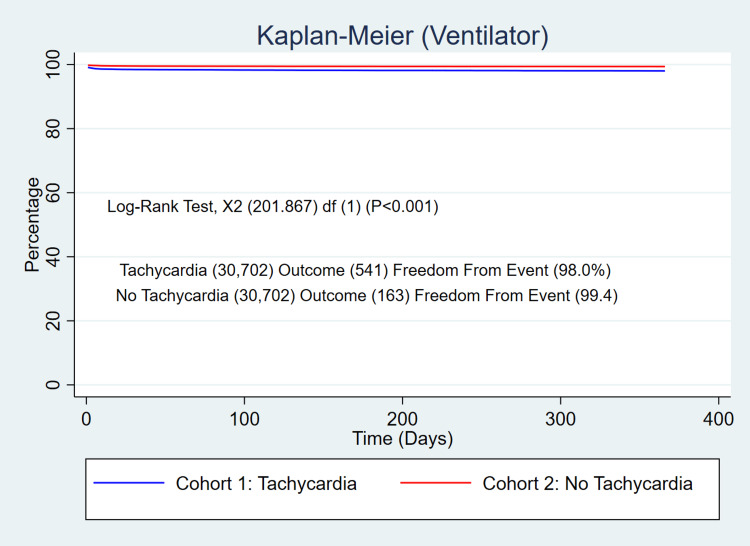
Risk of the use of a ventilator in patients with persistent tachycardia after SARS-COV-2 infection compared with the SARS-COV-2 control group without persistent tachycardia after PSM. Log-rank test: p<0.001 PSM, propensity score matching

The 365-day mortality was significantly higher in patients who developed persistent tachycardia after SARS-COV-2 infection (5.1% vs 2.1%, p<0.001) with a relative risk (RR) of 2.44 (95% CI 2.23-2.67, p<0.001) (Figure 1). The patients with persistent SARS-COV-2 tachycardia also exhibited a significant increase in the use of critical care services (5.8% vs 2.2%, p<0.001) with a RR of 2.64 (95% CI 2.41-2.88, p<0.001) (Figure 2), and ventilator utilization (1.8% vs 0.5%, p<0.001) with a RR of 3.32 (95% CI 2.79-3.95, p<0.001) (Figure 3).

Out of the 30,705 patients who developed persistent tachycardia after SARS-COV-2 infection, 22,878 (74.5%) had documentation of whether they received a complete vaccination series; 8,038 (35%) were vaccinated, while 14,840 (65%) were not vaccinated or were only partially vaccinated. After PSM (Table 2), the patients without complete vaccination who developed persistent tachycardia after SARS-COV-2 infection had higher rates of mortality (5.9% vs 2.3%, p<0.001). These patients also had a higher utilization of critical care services (8.3% vs 3.6%, p<0.001) and higher ventilator utilization (3.8% vs 0.6%, p<0.001) compared to completely vaccinated patients.

**Table 2:** Baseline characteristics of vaccinated and non-vaccinated patients with tachycardia BMI, body mass index; CAD, coronary artery disease; COPD, chronic obstructive pulmonary disease; TIA, transient ischemic attack

	Before matching			After matching		
	COVID-19 tachycardia + vaccine (n=8,038)	COVID-19 tachycardia + no vaccine (n=14,840)	P-value	COVID-19 tachycardia + vaccine (n=7,442)	COVID-19 tachycardia + no vaccine (n=7,442)	P-value
Age at index						
Mean ± SD	46.9 ± 17.9	43.2 ± 16	0.14	45.7 ± 17.6	44.7 ± 16.5	0.82
White	60.9%	57.5%	0.07	60.9%	60.9%	0.95
Female	56.9%	54.7%	0.04	56.9%	56.9%	0.99
Male	43.1%	43.7%	0.01	43.1%	43.1%	1.00
Hispanic	14.5%	9.5%	0.15	14.5%	14.5%	0.88
African American	1.8%	2.3%	0.03	1.8%	1.8%	0.83
Asian	2.4%	1.7%	0.01	2.3%	2.3%	1.00
Comorbidities (%)						
Hypertension	40.1%	21.53%	0.41	40.1%	40.2%	0.86
CAD without angina	24.5%	10.7%	0.37	24.5%	24.6%	0.96
Heart failure	20.1%	7.7%	0.36	20.0%	20.1%	0.92
COPD	8.6%	3.0%	0.24	8.6%	8.5%	0.59
Diabetes mellitus	7.1%	2.3%	0.23	7.1%	7.1%	0.80
Nicotine dependence	6.9%	3.7%	0.14	6.9%	7.01%	0.70
History of TIA	1.17%	0.7%	0.05	1.2%	1.1%	0.30
BMI greater than 35%	58.3%	33.4%	0.10	58.3%	58.3%	0.94

## Discussion

Sinus tachycardia is classified as a heart rate greater than 100 bpm [8]. Physiological processes, such as exercise, stress, or anxiety, can cause sinus tachycardia. However, tachycardia can also be a response to an ongoing pathological process of either cardiac or non-cardiac etiology. The human body's response to infections, hypoxia, anemia, and shock are all common causes of pathological responses that result in sinus tachycardia [9]. The majority of these are reversible with the resolution of the underlying cause.

In this retrospective cohort of COVID-19 patients, we demonstrated the persistence of sinus tachycardia more than 12-16 weeks post-infection with a prevalence of 2.2%. Persistent sinus tachycardia after infection has been documented in patients with various coronavirus strains, however, with much higher rates. The first study mentioning this possibility was by Yu and colleagues, who examined patients with SARS-COV-1 (SARS) coronavirus strains in 2006 [10]. At that time, they noted a high prevalence of tachycardia during the initial infection. However, they also observed that a tachycardic response remained in nearly 40% of patients (out of a cohort of 121) beyond three weeks post-infection. The shorter timeframe of follow-up in this study may account for the higher prevalence. Aranyo and colleagues found a prevalence of 20% in post-COVID-19 patients (40 out of 200 patients) [4]. However, it is important to note that their study defined tachycardia as a mean 24-hour heart rate above 90 bpm. In our study, we used an average heart rate greater than 100 bpm, potentially explaining the lower prevalence in our study.

In our study, hospitalized patients who developed persistent tachycardia after SARS-COV-2 infection had a higher prevalence of comorbidities. After PSM for key comorbidities and demographic factors, these patients were discovered to have two to three times increased mortality, need for critical care services, and ventilator utilization compared with patients who did not develop persistent tachycardia. To our knowledge, this is the first study to compare the rates of healthcare utilization and mortality in SARS-COV-2 patients who suffer from post-COVID-19 tachycardia with that of patients without sinus tachycardia. Yu and colleagues investigated patients with SARS-COV-1 who suffered from cardiovascular complications including tachycardia; they did not note any correlation with admission to the ICU [10]. This may be due to differences in the virus, a smaller sample size, and a high prevalence of tachycardia of more than 70% in their study.

Amidst increased vaccinations worldwide, approximately 53% of the world's population completed the initial series of vaccinations by February 15, 2022 [1]. Our study found that among hospitalized patients who experienced persistent post-COVID-19 tachycardia, 35% were completely vaccinated while 65% were not. The unvaccinated SARS-COV-2 tachycardia group had markedly higher mortality, use of critical care services, and ventilator utilization. While prior studies have not specifically examined this particular cohort of post-COVID-19 tachycardia patients, our results are consistent with prior studies demonstrating decreased critical care admission for vaccinated individuals. However, results regarding differences in patient mortality have been inconsistent [11,12]. 

Our study does have several limitations to note. Due to its retrospective nature, the findings are subject to the inherent limitations of retrospective observational studies. These include selection bias and differences in care-seeking behaviors. The accuracy of the results depends on the coding accuracy and completeness of this database. Granular data on the need for critical care services with respect to specific types of critical care utilization are not available outside of billing codes recorded in the database. The overall mortality rate in this database is higher than the case fatality rate of COVID-19 infection in some countries, such as the United Kingdom and Italy. However, this is likely due to the selection bias associated with the inclusion of a large percentage of hospitalized patients with COVID-19 and the multinational nature of the database. Furthermore, documentation of vaccination status is limited by reporting accuracy and could have been biased with both under-reporting and over-reporting in some circumstances. Our cohort also included some patients prior to the availability of vaccination in December 2020 and does not differentiate between the variants of SARS-COV-2.

Finally, our study does not evaluate the impact of treatment on these outcomes, whether aimed at the viral infection itself or the persistent tachycardia. Studies have postulated that persistent tachycardia is attributed to dysregulation of the autonomic nervous system or cardiopulmonary deconditioning including beta-adrenergic hypersensitivity and brainstem dysregulation [4,5]. Inappropriate sinus tachycardia unrelated to viral infections demonstrates a similar underlying mechanism as those proposed for tachycardia as a result of COVID-19 [13]. The use of negative chronotropic medications, such as carvedilol or ivabradine, has been shown to effectively control post-COVID-19 tachycardia [13,14]. Outcomes such as mortality and healthcare utilization have not been evaluated as a targeted intervention in this population. We always recommend starting the discussion with a summary of the findings and have moved material originally later in the discussion to create this summary. 

## Conclusions

Using a large-federated database, we found that the prevalence of persistent tachycardia after SARS-COV-2 infection is notable at 2.2%. While this is considerably lower than early reports in the literature, the number of affected patients is substantial with over 700 million SARS-COV-2 cases diagnosed worldwide. Even one year after the inciting viral disease, patients with persistent tachycardia had a higher mortality rate and demonstrated greater healthcare utilization than those without persistent tachycardia indicating a significant healthcare burden worldwide. Individuals without complete vaccination who developed sinus tachycardia after SARS-COV-2 infection had poorer outcomes one-year post-infection than vaccinated individuals as well, indicating that vaccination is likely protective in this context. Further study should be conducted to evaluate the modifiable risk factors and potential effects of interventions in this population.
